# Haematological adaptations to High Intensity Interval Training (HIIT) in temperate and hot environments

**DOI:** 10.1186/2046-7648-4-S1-A145

**Published:** 2015-09-14

**Authors:** Ashley P Akerman, Samuel JE Lucas, Chris J Baldi, Rajesh Katare, James D Cotter

**Affiliations:** 1 School of Physical Education, Sport and Exercise Sciences, University of Otago, Otago, New Zealand; 2 Department of Physiology, University of Otago, Otago, New Zealand; 3 School of Medicine, University of Otago, Otago, New Zealand; 4 School of Sport, Exercise, and Rehabilitation Sciences, University of Birmingham, UK

## Introduction

Blood volume is one determinant of cardiorespiratory power and capacity, and is believed to play a role in health and disease. Blood volume, and its components - plasma volume (PV) and red cell volume (RCV) - increase modestly in response to either endurance training or High Intensity Interval Training (HIIT)[1]. Plasma volume increases more rapidly than red cell mass[2] with exercise training, and increases to greater extent with the addition of environmental heat stress. It remains unclear whether PV remains elevated over several weeks of progressively-incrementing exercise in the heat, and also whether RCV is also expanded more by exercise in the heat than by exercise alone. The aim of this study was to investigate the haematological responses to progressive HIIT undertaken with and without additional heat stress.

## Methods

In a randomised crossover design, 10 sedentary participants (5 female; 33 ± 9 y; 189.4 ± 9.1 cm; 82.5 ± 15.0 kg; 38 ± 5 mL.kg^-1^.min^-1^) completed two 8-wk training regimes of HIIT (5 d.wk^-1 ^4 × 4 min aerobic cycle ergometry progressing to 90-95% HR_MAX, _supplemented with whole-body resistance band exercises, for 30-60 min per session), separated by 8-wk washout. One regime was in temperate (EX+TEMP; 23 ± 2°C, 36 ± 5% RH) and one in hot (EX+HEAT; 40 ± 0°C, 61 ± 2% RH) conditions. Blood volume, PV and RCV were measured at baseline, 4 wk and 8 wk, using carbon monoxide rebreathing after 15-min seated rest. Data were analysed using linear mixed method modelling.

## Results

Preliminary analysis indicates that despite no significant time or interaction effects for BV or any of its components, BV was higher on average for the HEAT+EX than TEMP+EX regime (74.0 ± 3.5 vs. 71.5 ± 2.6 mL.kg^-1^, respectively; P = 0.047). RCV followed the same pattern (higher in HEAT+EX vs. TEMP+EX by 1.1 ± 0.8 mL.kg^-1^; 95% CI: 0.4 - 1.9 mL.kg^-1^; P = 0.009). In absence of a time effect on RCV, haematocrit was used to determine PV change. Plasma volume was expanded (P = 0.014) only slightly at 4 weeks (by 2.5 ± 2.1% in HEAT+EX; CI: 1.0 - 3.9%; and by 1.4% ± 2.33 in TEMP+EX; CI: -0.2 - 3.0%), to similar extent between regimes (HEAT - TEMP: 1.0%; CI: -1.2 - 3.2%). This expansion remained at a comparable level at 8 wk.

## Discussion and conclusion

HIIT conferred only small increases in plasma volume when measured at 4 and 8-wk, and no measured increase in RCV. HIIT performed in hot conditions provided no measurable additional increase in PV or RCV over HIIT performed in temperate conditions. Variability in CO-derived haematological measures was high (CV: 10.7 ± 1.9 %) and likely contributed to the lack of significant effects. However, it should also be noted that the volume of HIIT was high (5 d/wk at 90-95% HRmax), and the relative exercise intensity (%HR_MAX_) was matched between regimes, which may have minimised heat-induced effects *per se*.
